# Aging potentially reduces CD169 expression in sinus macrophages of pelvic lymph nodes

**DOI:** 10.1007/s00795-025-00433-3

**Published:** 2025-03-24

**Authors:** Yuki Ibe, Yojiro Ozaki, Toshiki Anami, Hiromu Yano, Yukio Fujiwara, Hidekazu Nishizawa, Ryoma Kurahashi, Takanobu Motoshima, Yoji Murakami, Junji Yatsuda, Yoshihiro Komohara, Tomomi Kamba

**Affiliations:** 1 Department of Cell Pathology, Graduate School of Medical Sciences, Kumamoto University, 1-1-1, Honjo, Kumamoto Chuo-Ku, Kumamoto, 860-8556 Japan; 2 Department of Urology, Graduate School of Medical Sciences, Kumamoto University, Kumamoto, Japan; 3 Center for Metabolic Regulation of Healthy Aging, Kumamoto University, Kumamoto, Japan

**Keywords:** Prostate cancer, CD169, CD3, CD8, CD103, Sinus macrophages

## Abstract

The number of patients with prostate cancer has been increasing around the world. Although anticancer immunotherapy targeting the immune checkpoint molecules has been approved for many types of cancer, no significant anti-cancer effects have been observed in patients with prostate cancer. Lymph node sinus macrophages (LSMs) are known to work as antigen-presenting cells, which are critical for anticancer immune responses. Previous studies have suggested that CD169 expression in LSMs affects anticancer immune responses in several cancers, including prostate cancer. In the present study, we aimed to examine the correlation between the tumor immune microenvironment and activation status of LSMs in patients with prostate cancer. Forty-two cases of high-risk localized prostate cancer treated using robot-assisted laparoscopic radical prostatectomy and lymph node dissection between 2017 and 2021 were enrolled. CD169 expression in LSMs was examined by immunohistochemistry. The results indicated that CD169 expression in LSMs was significantly decreased in older (≥ 75 years) compared with younger patients. However, no significant correlation was found between CD169 expression and any other clinicopathological factors. In addition, CD3- and CD8-postitive lymphocytes in primary cancer tissues were evaluated in the same cases, and their correlations with CD169 expression in LSMs were tested. Although these lymphocytes tended to be higher in CD169^high^ than in CD169^low^ cases, the difference was not statistically significant. In conclusion, we found that CD169 expression was upregulated in older patients and tended to be related to T cell infiltration in cancer tissues. Therefore, the downregulation of CD169 in LSMs might be involved in the reduced anticancer immune response in prostate cancer.

**Supplementary Information:**

The online version contains supplementary material available at 10.1007/s00795-025-00433-3.

## Introduction

Over the past 40 years, the incidence of prostate cancer in Japan has increased approximately 30-fold, making it one of the leading cancers among men, alongside lung, stomach, and colorectal cancers. In terms of site-specific cancer incidence, prostate cancer ranks first among male cancers in Japan. In addition, in 2019, the number of deaths due to prostate cancer was 12,544, accounting for 5.7% of all male cancer deaths (total male cancer deaths in 2019: 220,339), making it the sixth leading cause of cancer-related deaths in men [1].

The primary treatment modalities for prostate cancer include surgery, radiation therapy, active surveillance, hormone therapy, and chemotherapy. Surgical treatment is performed for patients with low- to high-risk localized prostate cancer [2, 3]. Lymph node (LN) dissection is not generally performed in low-risk cases, but is recommended in intermediate- and high-risk cases. Immunotherapy targeting immune checkpoint molecules has been approved in several cancers, including lung cancer, gastrointestinal cancer, and kidney cancer [4]. Although a previous case report documented the significant anticancer effects of immunotherapy for advanced prostate cancer with the microsatellite instability-high phenotype [5], prostate cancer has been considered to be resistant to immunotherapy because of its low immunogenicity.

LNs are a secondary immune organ that play important roles in the cancer immune cycle [6]. The deletion of LNs in animal models has been shown to suppress anticancer immune responses significantly [7], which suggests that antigen-presenting cells such as dendritic cells and macrophages in regional LNs engulf antigens or debris refluxed from peripheral cancer tissues and activate antigen-specific T lymphocytes in LNs. Sialoadhesin (CD169/Siglec-1), located on chromosome 20p13, is a member of the sialic acid-binding immunoglobulin-like lectin (Siglec) family. It is predominantly expressed in sinus macrophages in lymph nodes (LNs) and serves as a key marker for lymph node sinus macrophages (LSMs). CD169 plays an important role in immune surveillance by mediating the recognition and uptake of sialylated antigens. Animal studies using transgenic mice, which deplete CD169-expressing cells by diphtheria toxin, have demonstrated that LSMs can engulf dead cancer cells and activate cancer antigen-specific cytotoxic T lymphocytes through cross-presentation [8].

Strömvall et al. [9] showed that CD169 expression was decreased in pre-metastatic LNs in a rat metastatic prostate cancer model, and that reduced CD169 expression in LSMs was associated with worse cancer-specific survival in human prostate cancer.

Therefore, several studies have demonstrated the significance of CD169 expression in LSMs in the immune responses in prostate cancer; however, few studies have investigated the correlation between LSMs and the immune microenvironment in prostate cancer. Given this background, in the present study, we analyzed the relationship between CD169 expression in LSMs and immune cell infiltration in the primary tumor using resected samples.

## Materials and methods

### Prostate cancer and lymph node samples

This retrospective study used clinical information and paraffin-embedded samples obtained from 42 patients with high-risk localized prostate cancer who underwent treatment with robot-assisted laparoscopic radical prostatectomy and LN dissection between 2017 and 2021 at Kumamoto University Hospital (Kumamoto, Japan). Data collection and analysis were approved by the Institutional Review Board of Kumamoto University (#2059). Tissue samples were fixed with 10% neutral-buffered formalin and embedded in paraffin.

### Immunohistochemistry (IHC)

Sections (thickness, 3 µm) were immersed in EDTA solution (pH 8.0) and heated in a pressure cooker for antigen retrieval. The following primary antibodies were used: anti-NKX3.1 antibody (clone EP356; Nichirei, Tokyo, Japan), anti-CD169 antibody (clone SP216; Abcam, Cambridge, UK), anti-CD163 antibody (clone 10D6; Novocastra, Deer Park, IL, USA), anti-CD3 antibody (clone SP7; Nichirei), anti-CD8 antibody (clone C8/144B; Nichirei), and anti-CD103 antibody (clone EPR4166; Abcam). NKX3.1 is a prostate-specific homeobox gene regulated by androgens [10]. It plays a crucial role in maintaining prostate tissue integrity and is often used as a marker to identify prostate cancer cells. Samples were incubated with peroxidase-labeled goat anti-mouse or rabbit secondary antibodies (anti-mouse, #424,132, and anti-rabbit, #424,142; Histofine, Nichirei Biosciences, Tokyo, Japan). Immunoreactions were visualized using a diaminobenzidine substrate kit (#425,011; Nichirei Biosciences). Cell counting was performed using HALO software (Indica Labs, Albuquerque, NM, USA).

### Multiplex immunohistochemistry

Anti-CD3 antibody, anti-CD8 antibody, and anti-CD103 antibody were used for multiplex immunohistochemistry. Immune reactions were visualized using aminoethyl carbazole substrate solution (Nichirei). Stained slides were digitally scanned using Nanozoomer S20 (Hamamatsu Photonics, Shizuoka, Japan), and destaining and antibody stripping were performed. The sections were then re-stained, and the scanning, destaining, and antibody stripping were repeated. Color resolution into pseudo-fluorescent images and fusion of each image were performed using Halo software to create multichannel pseudo-fluorescent images.

### Statistical analyses

Statistical analyses were carried out using GraphPad Prism 9 (GraphPad Software, San Diego, CA, USA). We used the Mann–Whiney *U* test and the χ^2^ test for comparisons in this study. For all analyses, differences were considered to be statistically significant at *p* < 0.05.

## Results

### Micrometastasis was found in two of 36 cases initially diagnosed as no lymph node metastasis

**Fig. 1:**
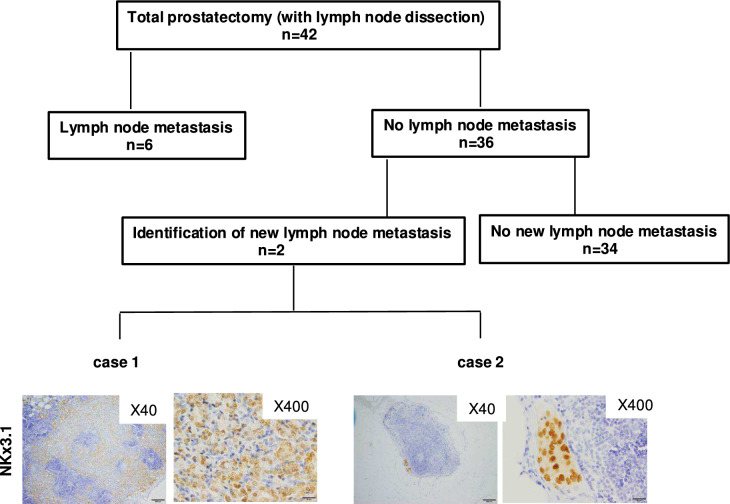
Identification of micrometastasis in pelvic lymph nodes. Two images of immunohistochemical staining for NKX3.1 in pelvic lymph nodes with micrometastasis are presented (×40 and ×400)

The characteristics of the 42 cases enrolled in the present study are presented in Table 1. Among the 42 cases, six were found to have LN metastasis upon routine pathological examination, while 36 cases were diagnosed as having no LN metastasis. We tried to examine the micrometastasis in the LNs (a total of 845 LNs in 36 cases) by means of IHC using anti-NKx3.1 antibody. Of the 36 cases without LN metastasis, micrometastasis was newly identified in two cases (Fig. 1).

**Table 1:** Patients’ characteristics

	(*n* = 42)
Median age	70.5
	(56–79)
Initial PSA(ng/ml)	12.715
	(1.65–69.91)
*Clinical T stage*	
cT1c	15
cT2a	17
cT2b	4
cT2c	5
cT3a	1
*Pathological T stage*	
pT2a	2
pT2b	0
pT2c	16
pT3a	14
pT3b	10
*Pathological N stage*	
pN0	36
pN1	6
*Gleason score*	
7	26
8	8
9	7
10	1
*PSA failure*	
Yes	29
No	13

### CD169 expression in LSM was elevated in older patients, but not associated with any other clinicopathological factors, including LN metastasis

**Fig. 2:**
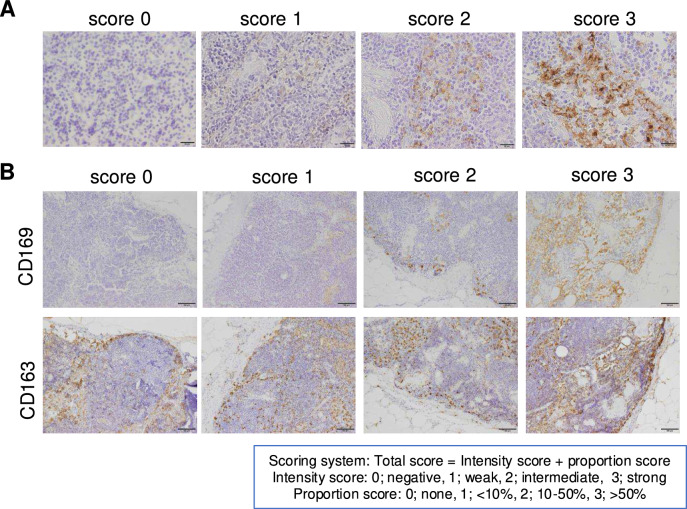
Scoring of CD169 expression in LSMs. **A** The intensity score is classified into four levels: 0 (negative), 1 (weak positive), 2 (moderate positive), and 3 (strong positive). **B** Representative images of immunostaining for CD169 and CD163. While CD163 expression is consistently observed, the expression of CD169 varies among cases. The proportion score was categorized as 0 (none), 1 (< 10%), 2 (10–50%), and 3 (> 50%). The total score was calculated as the sum of the intensity and percentage scores

**Fig. 3:**
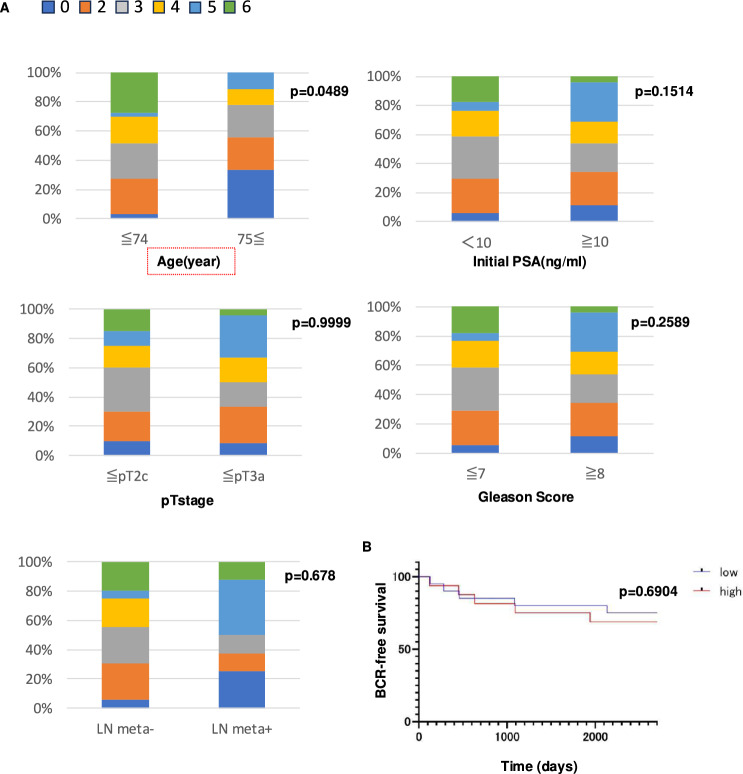
Correlation between CD169 expression and clinicopathological factors. **A** CD169 total scores were classified into low (≤ 3) and high (≥ 4) groups. Statistical analysis revealed a significant association between CD169 expression and patient age, but no significant correlation with PSA, pT stage, Gleason score, or lymph node metastasis. **B** Kaplan–Meier analysis of BCR-free survival showed no significant difference between the low and high CD169 score groups

In the next step, CD169 expression in LSMs was tested by IHC. All LSMs were positive for CD163 (Fig. 2A). The intensity of CD169 positivity was classified by score into four groups: 0 for negative, 1 for weak, 2 for moderate, and 3 for strong (Fig. 2A). The distribution of CD169 expression was also classified by score into four groups by means of positive staining area: 0 for no positive cells, 1 for < 10%, 2 for 10%–50%, and 3 for > 50% (Fig. 2B). The combination of the distribution and intensity scores was defined as the total CD169 score. The total CD169 score was then classified into two groups: low (≤ 3 points) and high (≥ 4 points). Then the statistical association was investigated between the CD169 expression score in LSMs and clinicopathological factors. The clinicopathological factors analyzed included age, initial prostate-specific antigen (PSA) levels, pathological T stage, Gleason score, and the presence or absence of lymph node metastasis determined by pathological diagnosis. The classification criteria for each factor were as follows. Age was divided into two groups: elderly patients (≥ 75 years) and non-elderly patients (≤ 74 years). Initial PSA levels were categorized according to the National Comprehensive Cancer Network (NCCN) guidelines into a low-risk group (< 10 ng/mL) and a medium/high-risk group (≥ 10 ng/mL). The pathological T stage was classified based on NCCN guidelines as a low/medium-risk group (≤ pT2c) and a high-risk group (≥ pT3a). Gleason scores were also categorized according to NCCN guidelines into a low/medium-risk group (≤ 7) and a high-risk group (≥ 8). These criteria were used for subsequent analysis. As shown in Fig. 3A, higher CD169 scores were observed in cases with a younger age; however, no other factors, including initial prostate-specific antigen (PSA), pathological T stage, Gleason score, and LN metastasis, were associated with CD169 scores. In addition, no correlation was found between CD169 expression and biochemical recurrence (BCR) (Fig. 3B).

### A potential positive association was observed between CD169 expression in LSMs and infiltrating lymphocytes in prostatic primary lesions

**Fig. 4:**
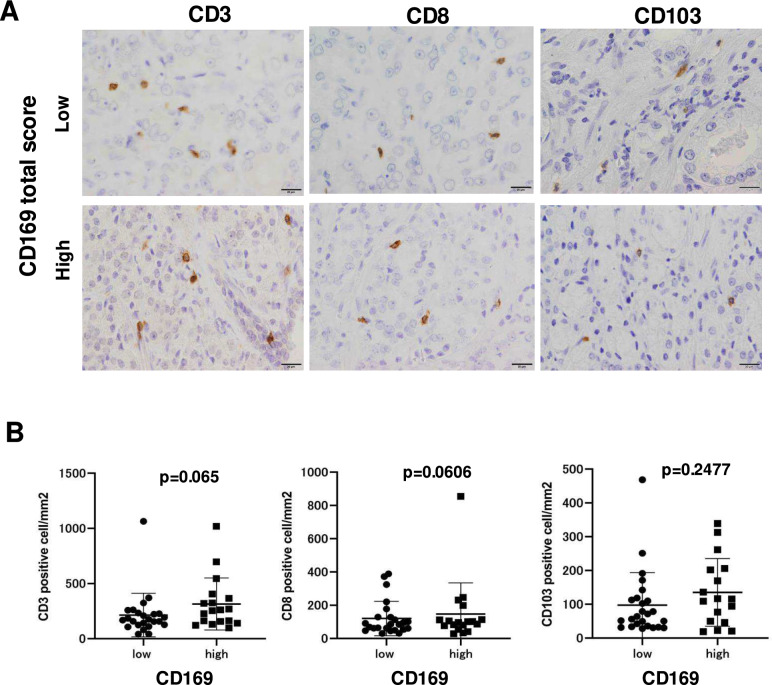
Correlation between CD169 expression in LSMs and T cell infiltration in primary lesions. **A** Representative images of IHC with CD3, CD8, and CD103 in primary prostate cancer tissues are shown for cases with low and high CD169 scores. **B** Cells were counted and compared between the low and high CD169 score groups

At the final step, T cell infiltration in prostatic primary lesions was examined by IHC with CD3, CD8, and CD103, positive cells were counted (Fig. 4A), and their correlations with CD169 scores in LSMs were investigated. CD3- and CD8-positive T lymphocytes seemed to be increased in cases with a high CD169 score, but did not reach the level of statistical significance (Fig. 4B). Furthermore, the correlations between the density of T lymphocytes and clinicopathological factors were tested. However, no significant differences were observed for any of the parameters (Supporting Figs. 1 and 2).

**Fig. 5:**
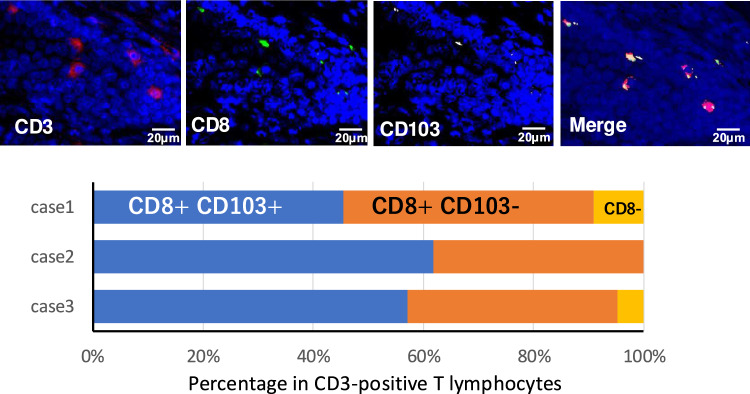
The proportion of CD103-positive T cells among CD8-positive cells (TRM) was analyzed using multiple immunohistochemistry (IHC) techniques. It was observed that more than 96% of CD3-positive T cells (average across 3 cases) were CD8 positive, and among these, 54% (average across 3 cases) expressed CD103

Recent studies have indicated the significance of the CD103-positive tissue-resident memory phenotype of CD8-positive T cells (TRM cells) in the tumor microenvironment (TME) [11]. IHC with CD103 was additionally performed and positive cells were counted. However, the number of CD103-positive cells was not associated with CD169 expression (Fig. 4A and B). Although the number of CD103-positive cells and ratio of CD103-positive cells in CD3-positive cells were significantly increased in the lower compared with the higher Gleason score group (Supporting Fig. 3), no correlations were found between CD103-positive cells and any other clinicopathological factors. We tested the percentage of CD103-positive T cells in CD8-positive cells (TRM) by means of multiple IHC technique. As shown in Fig. 5, more than 96% (average of 3 cases) of CD3-positive T cells were CD8 positive and 54% (average of 3 cases) of CD8-positive T cells were positive for CD103.

## Discussion

In the first step of this study, we tested the incidence of micrometastasis that could not be detected by routine pathological examination using hematoxylin and eosin-stained sections by means of IHC. Micrometastasis was newly found in two of 36 cases (5.6%). Maxeiner et al. [12] previously tested for micrometastasis by IHC using 2352 LNs in 193 patients with prostate cancer and found new micrometastasis in 17 (8.8%). They also reported that micrometastasis was the strongest predictor of BCR; therefore, they described that IHC seems to have high diagnostic value for the detection of micrometastases in initially nodal-negative patients.

Next, we focused on the expression of CD169 in LSMs and its immunological significance in relation to clinicopathological factors. Our findings demonstrated an age-associated reduction in CD169 expression. Although this change was not statistically significant, increased infiltration of CD3- and CD8-positive T lymphocytes in primary lesions was found in cases with a high CD169 score. These observations suggested the potential impairment of pelvic LN immune function in older individuals. Notably, this reduction was independent of other clinicopathological factors, including LN metastasis. Age-related changes in LSMs are known to have a significant impact on immunoregulation within the LN microenvironment. Kanemitsu et al. [13] reported an aging-related decline in sinus macrophages in mesenteric LNs, implicating this reduction in the diminished functional capacity of LNs. Such a decrease in sinus macrophages is thought to compromise antigen uptake and T cell activation, which are critical for immune surveillance. Our findings align with this, suggesting that reduced CD169 expression in pelvic LNs among older individuals may follow a similar mechanism. The pelvic and mesenteric lymph nodes share common immunological functions but differ anatomically and in their exposure to specific antigens such microbiota. Future studies should further explore these regional differences.

Because recent studies have indicated the significance of CD8-positive T cells with the CD103-positive tissue-resident memory phenotype (T_RM_ cells) in the TME, IHC for CD103 was also performed in all cases. As a result, we found that approximately 50% of the T lymphocytes were T_RM_ cells. CD103, which is also known as integrin αE/β7, binds to the adhesion-binding protein E-cadherin and is present in peripheral tissues without secondary lymphoid organs [14]. In the present study, the number of CD103-positive cells was found to be significantly increased in the low Gleason score group. However, no other factors were associated with CD103-positive cells. As prostate cancer is known to be an immunologically “COLD” tumor [15], CD103 might not be as informative in prostate cancer.

In conclusion, the age-associated reduction observed in CD169 expression in LSMs may represent a key mechanism underlying immune evasion in prostate cancer. Furthermore, the findings by Kanemitsu et al. [13] suggest that the age-related decline in LSMs could be a contributing factor to systemic immune dysfunction and tumor progression. However, the limitations of this study include the relatively small sample size and the exclusion of pelvic lymph node samples from healthy individuals due to ethical considerations, which restricts our ability to clarify the relationship between decreased LSM and immune dysfunction in the elderly. Future studies should aim to elucidate the functional role of CD169-positive LSMs and explore age-specific therapeutic strategies, targeting these immune cells to enhance immunotherapy outcomes in older patients.

## Supplementary Information

Below is the link to the electronic supplementary material.

Supplementary file1 (PDF 1254 KB)

## Data Availability

The data in this manuscript will be shared by the corresponding author request.
