# Supplementary figures and images for: Aging potentially reduces CD169 expression in sinus macrophages of pelvic lymph nodes
Source: Med Mol Morphol. 2025 Mar 24;58(4):290–7. doi: 10.1007/s00795-025-00433-3 (PMC12644165; doi:10.1007/s00795-025-00433-3)

Supporting Figure 1

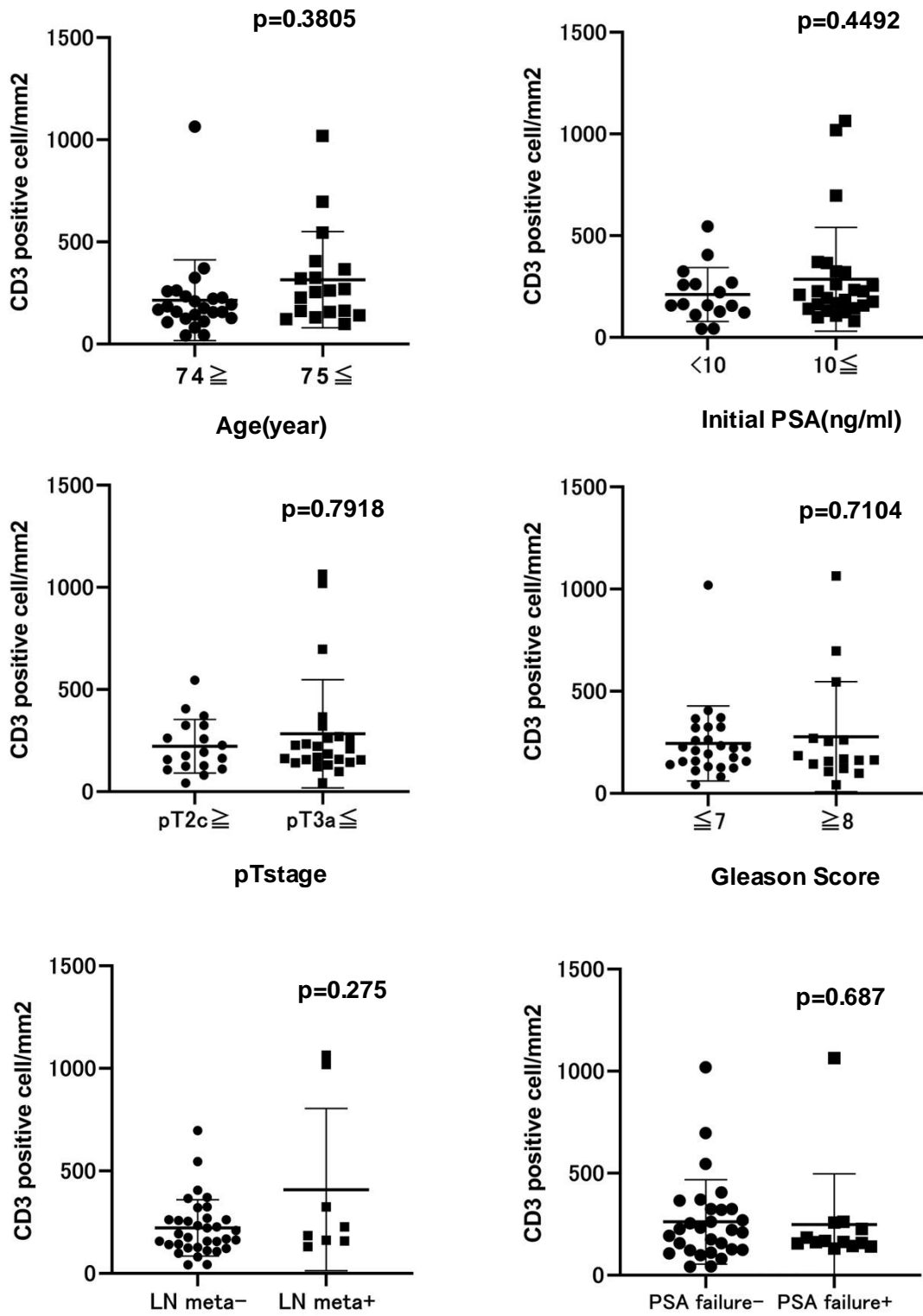

Supporting Figure 2

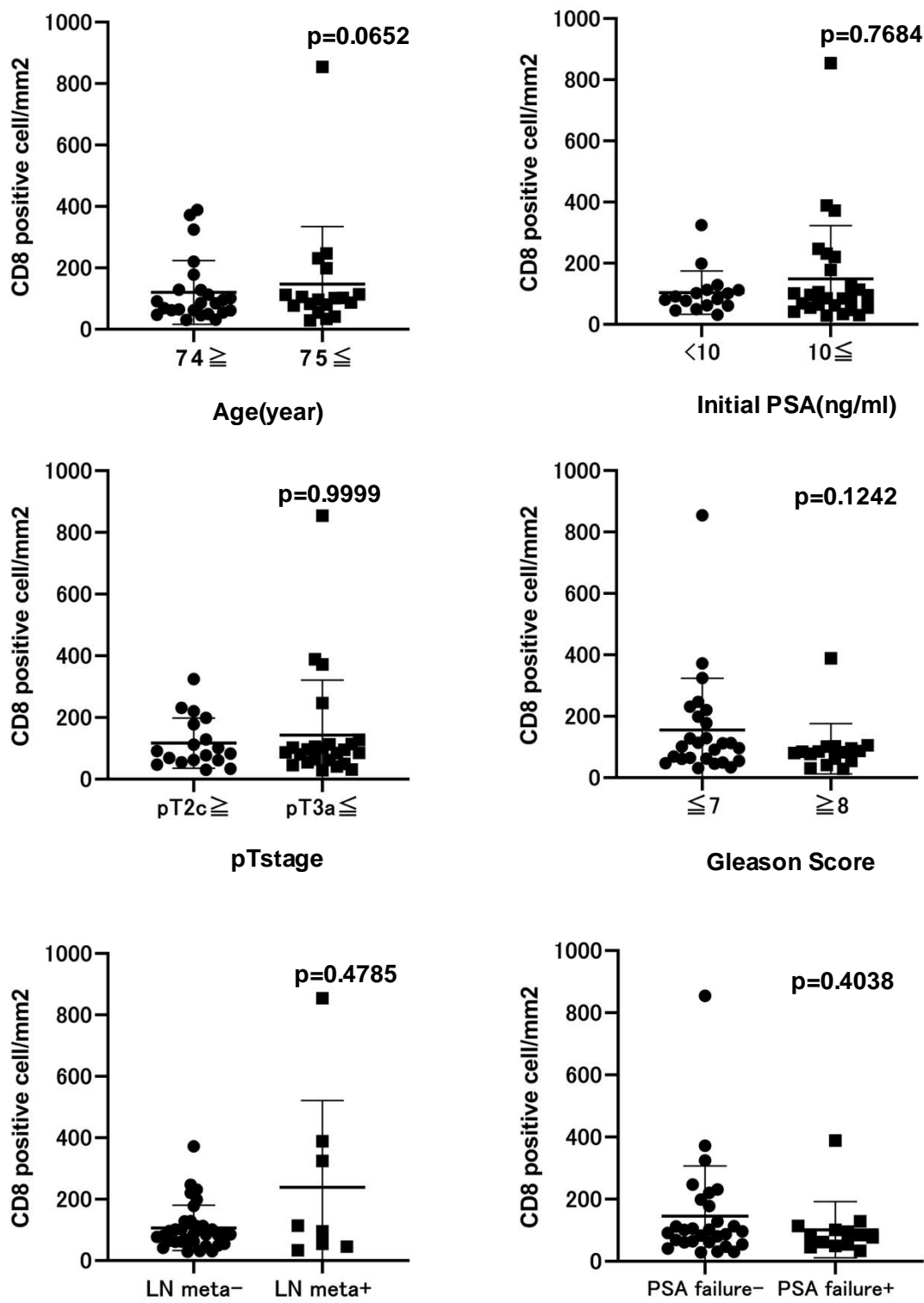

Supporting Figure 3

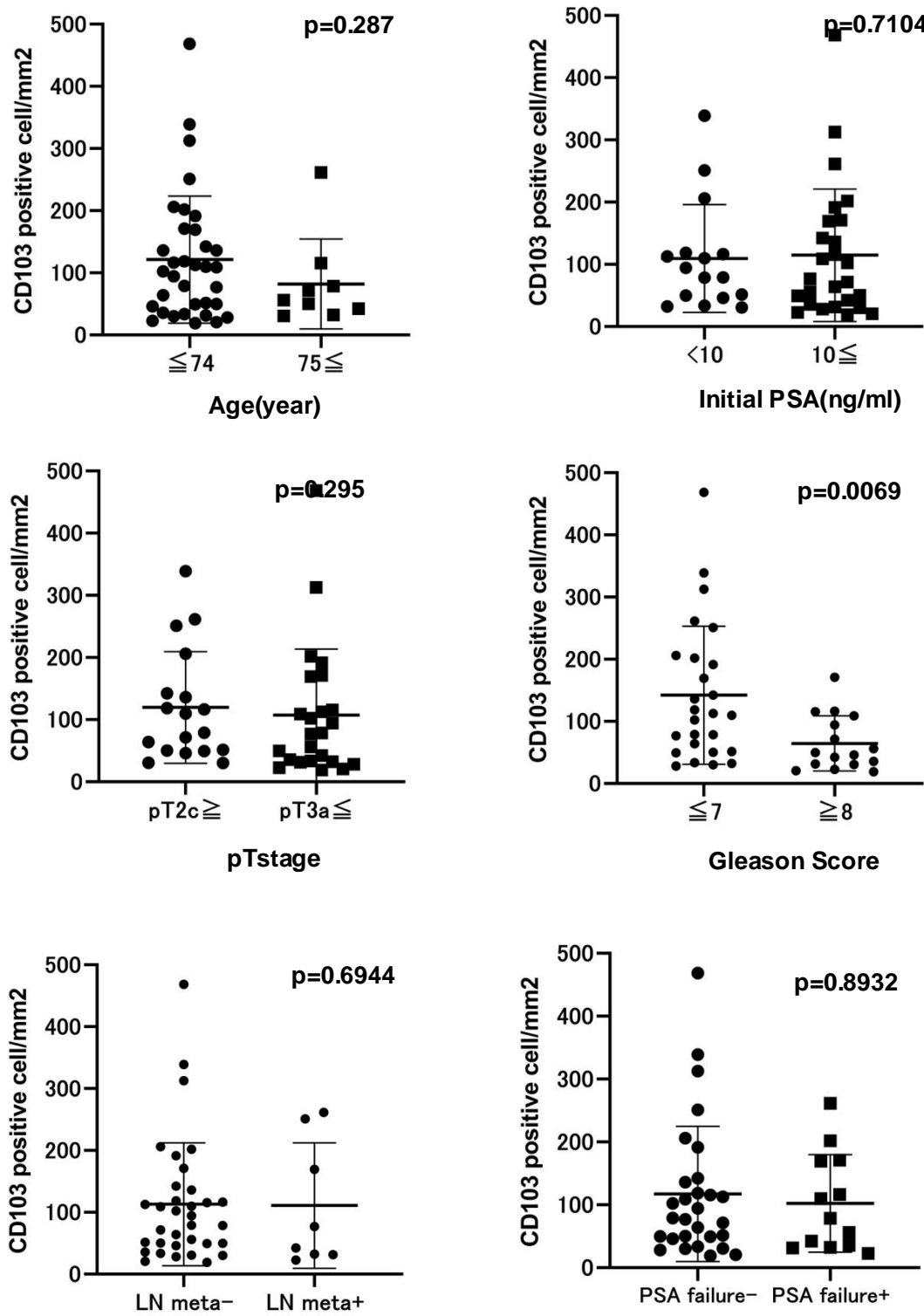

# Supporting Figure 4

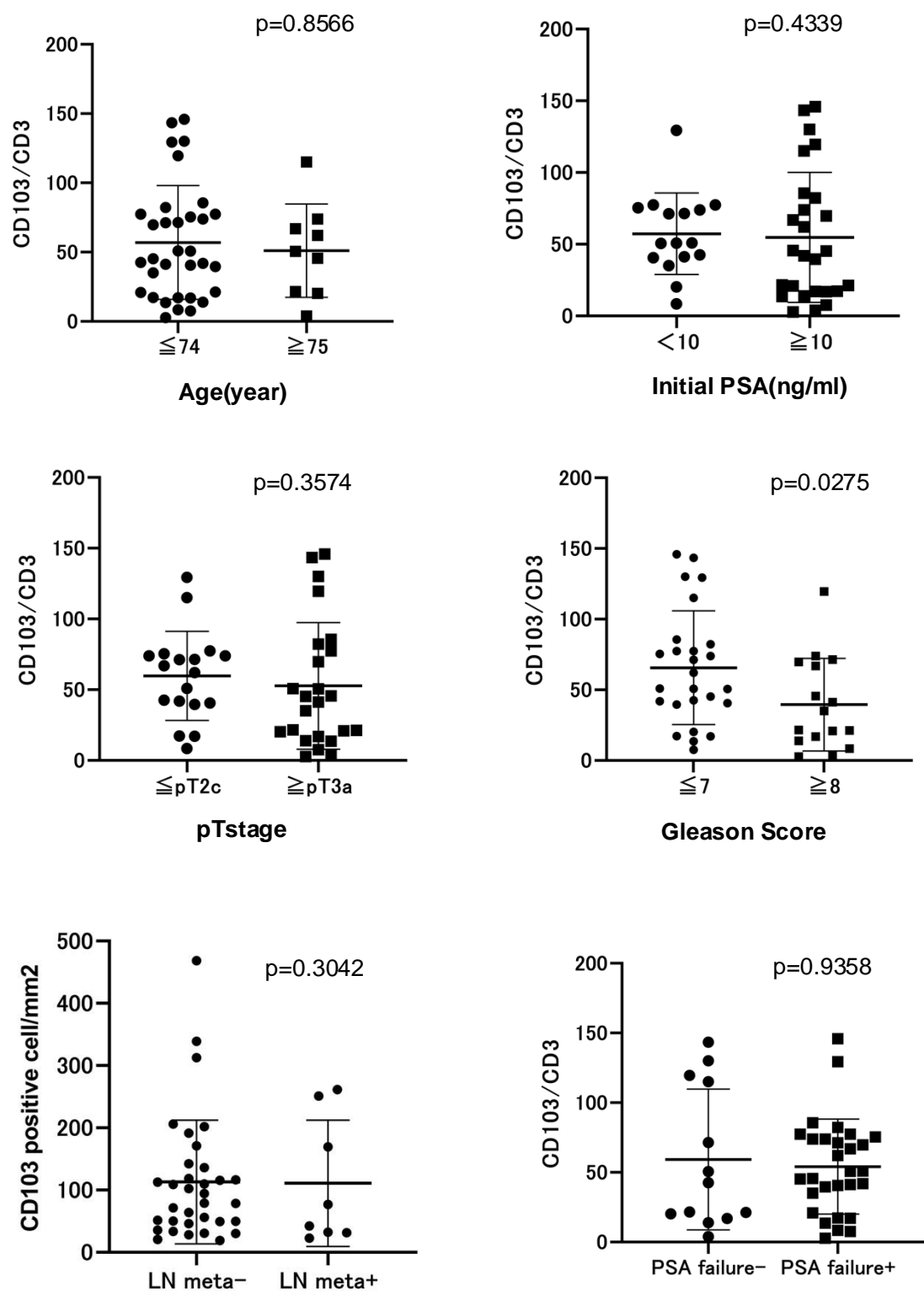

Supplement: Supplementary file 1 — Supplementary file1 (PDF 1254 KB) [file 795_2025_433_MOESM1_ESM.pdf]
